# Real-time forecast of temperature-related excess mortality at small-area level: towards an operational framework

**DOI:** 10.1088/2752-5309/ad5f51

**Published:** 2024-07-18

**Authors:** Malcolm N Mistry, Antonio Gasparrini

**Affiliations:** 1 Environment & Health Modelling (EHM) Lab, Department of Public Health Environments and Society, London School of Hygiene & Tropical Medicine, London, United Kingdom; 2 Department of Economics, Ca’ Foscari University of Venice, Venice, Italy

**Keywords:** temperature-related mortality, excess deaths, operational forecasting system

## Abstract

The development of innovative tools for real-time monitoring and forecasting of environmental health impacts is central to effective public health interventions and resource allocation strategies. Though a need for such generic tools has been previously echoed by public health planners and regional authorities responsible for issuing anticipatory alerts, a comprehensive, robust and scalable real-time system for predicting temperature-related excess deaths at a local scale has not been developed yet. Filling this gap, we propose a flexible operational framework for coupling publicly available weather forecasts with temperature-mortality risk functions specific to small census-based zones, the latter derived using state-of-the-art environmental epidemiological models. Utilising high-resolution temperature data forecast by a leading European meteorological centre, we demonstrate a real-time application to forecast the excess mortality during the July 2022 heatwave over England and Wales. The output, consisting of expected temperature-related excess deaths at small geographic areas on different lead times, can be automated to generate maps at various spatio-temporal scales, thus facilitating preventive action and allocation of public health resources in advance. While the real-case example discussed here demonstrates an application for predicting (expected) heat-related excess deaths, the framework can also be adapted to other weather-related health risks and to different geographical areas, provided data on both meteorological exposure and the underlying health outcomes are available to calibrate the associated risk functions. The proposed framework addresses an urgent need for predicting the short-term environmental health burden on public health systems globally, especially in low- and middle-income regions, where rapid response to mitigate adverse exposures and impacts to extreme temperatures are often constrained by available resources.

## 1 Introduction

Extreme weather phenomena such as cold spells and heat waves have been previously linked with substantial health burdens [1, 2], with the latter expected to become more frequent and intense due to climate change [3]. In addition, such health risks from thermal exposures are known to vary geographically due to variations in vulnerability linked with socio-economic, climatological, and infrastructural characteristics [4]. There is, therefore, a growing interest in establishing methodologies to forecast temperature-related health impacts in advance, contributing in the short term to the planning of timely national and local interventions, as well as in impact-based warning systems, in particular for heat [5, 6]. A systematic and comprehensive temperature-related risk prediction system for forecasting cold- and heat-related excess deaths at the local scale on appropriate lead times, could improve preparedness of regional health authorities and better allocation of vital and often constrained public health resources.

## 2 Real-time operational framework

### 2.1 Shortcomings of existing health warning systems

When quantifying the impacts of an environmental hazard on health, a commonly used metric in environmental epidemiology and public health is the ‘excess deaths’ or ‘excess mortality’. Defined as the number of deaths caused directly by an event or the number of deaths that would not have occurred in the absence of the same (e.g. a heatwave), excess deaths can be aggregated over space (across geographic areas) and time, as well as across different age groups. Total excess deaths is a measure frequently used to quantify the health burden associated with extreme events, as it encompasses various health risk pathways and is based on standardised and readily available statistics [7].

**Figure 1:**
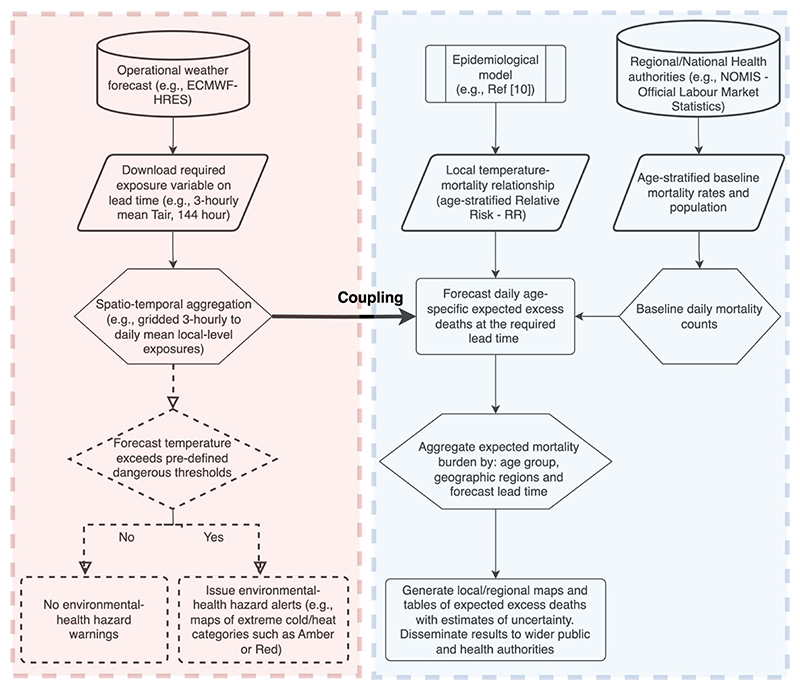
Schematic framework for forecasting temperature-related excess deaths in real-time. The red shaded block of the diagram indicates the meteorological hazard component of the framework that is currently operationally implemented by several national weather and health monitoring authorities, such as the UKHSA-UKMO in the UK and CDC-NOAA in the US. While these alerts issued to the wider population are important in highlighting the risks associated with exposures to both extreme cold and heat, they lack the detailed estimation of expected excess deaths that would require the coupling with the sophisticated epidemiological designs, historical data on mortality rates and population by age groups, and advanced statistical methods (blue shaded block). The output of the meteorological hazard component indicated by dashed lines within the red shaded block is not part of our proposed framework here, but can be easily included as additional hazard exposure maps (e.g. forecast maps of extreme temperature overlaid with current population exposed at local levels). The combined (coupled) workflow can be automated to forecast and monitor expected excess deaths from cold and heat, requiring minimal computational resources and publicly available tools discussed in the study.

Operational short-term temperature-related health advisories currently issued by national health and meteorological services, such as the: (i) weather-health Alert (WHA) system implemented jointly by the UK Health Security Agency (UKHSA, formerly known as Public Health England) and the UK Met Office (UKMO) in the United Kingdom [8, 9], and (ii) National Integrated Heat Health Information System (NIHHIS) developed by the Centres for Disease Control and Prevention (CDC) in collaboration with the National Oceanic and Atmospheric Administration (NOAA) in the United States (www.heat.gov/), come with certain limitations. While both WHA and NIHHIS, among others, are built as integrated information systems for providing actionable, science-based information to help protect people from exposure to cold and/or heat, their underlying frameworks are designed primarily around the ‘weather’ component of the event (shown by the red-shaded block in figure 1). Put differently, such WHA systems are well suited for issuing hazard warnings: while these can play a role in mitigating the detrimental impacts of temperature extremes on the exposed population, they fall short in anticipating the expected burden on the public health system. Specifically, the health advisories and alerts are not designed to quantify the associated location-specific health risks (for instance, expected excess deaths) and, more importantly, they are not designed to tailor the warning to local exposure level or differential vulnerability of the population. This would require a more thorough framework that incorporates information on high-resolution weather forecasts with area-specific risk functions to compute spatially-disaggregated health impact measures, thus differentiating the warning depending on the local characteristics.

### 2.2 Conceptual design of a new system

We present here a conceptual framework for the design and development of a real-time prediction of expected temperature-related excess deaths at small geographic scale (figure 1). In brief, the underlying principle entails coupling spatially-resolved meteorological variables from routine weather forecasts (here near-surface sub-daily mean air temperature, *T*_air_), with temperature-mortality association derived using state-of-the-art epidemiological designs and statistical methods. The coupling would enable the estimation of excess deaths attributable to specific exposures, such as cold or heat on lead times of the underlying weather forecast.

The additional block in the conceptual design (shown as a blue-shaded region in figure 1) complements the weather component of the forecasts with epidemiological data. Here, the health impact component is based on a modelling framework described in a previous analysis that was used to derive detailed maps of risks associated with heat and cold in England and Wales, accounting for local variation in vulnerability [10]. However, the same can be adapted to other modelling approaches catering to other geographical regions and various spatial scales. In fact, the biggest novelty of such a framework is that it can be deployed and automated in near real-time to generate maps of age-specific excess deaths on a continuous basis. Naturally, the real value of such a framework can be most appreciated during extremes in cold and heat, thereby allowing the public health services to forecast the impending health risks and the associated burden on public health infrastructures from harsh weather events on local populations.

### 2.3 Health impact forecast framework

The health impacts can be computed using established methods to quantify excess mortality associated with non-optimal temperature [10–13]. The calculation is based on three sources of information, namely: (i) estimated exposure-response relationships between temperature and mortality; (ii) forecasted temperatures during the study period; and (iii) baseline mortality counts. The assessment can be tailored to the available data regarding the geographical stratification (e.g. from region to small administrative areas) and optional age/sex grouping.

Exposure-response relationships can be estimated using distributed lag non-linear models (DLNMs) [11], allowing non-linear and lagged dependencies and therefore accounting for delayed effects and potential short-term mortality displacement. The estimated exposure-response curve can vary by age/sex and location, characterising the differential vulnerability to heat and cold across population sub-groups and geographical areas. For each day *t*, age/sex group *a*, and area *j*, we can compute the expected excess deaths *d*_*taj*_ as:

$$d_{taj}=\left(p_{aj}\times m_{aj}/365.25\right)\times \left(1-e^{\beta_{taj}}\right).$$

The quantities *p*_*aj*_ and *m*_*aj*_ are the population and baseline annual mortality rate for each age/sex group, respectively. These terms are multiplied and divided by 365.25 to recover the baseline daily deaths, assumed identical on each day *t*, or optionally accounting for seasonality. The term $e^{\beta_{taj}}$ represents the relative risk (RR) dependent on the temperature forecasted in day *t*, defined by the age/sex and area-specific exposure-response function using a reference corresponding to the minimum mortality temperature (MMT). The expected excess deaths can then be aggregated by larger geographical areas and periods. Note that, in the equation above, the term $1-e^{\beta_{taj}}$ corresponds to the traditional (RR-1)/RR representing the attributable fraction [14]. As a matter of interpretation, it is important to note that the computation of the excess mortality follows a forward perspective [12], whereby an exposure experienced in a given day causes a lagged increase in risk. Therefore, the expected excess deaths associated with the temperature forecasted on day *t* would occur not just on the same day, but also in the following days consistently with the lagged risk represented by the DLNM.

Uncertainty in the estimated expected excess mortality was quantified as empirical confidence intervals (eCIs) using Monte Carlo simulations, using a method previously described [12]. In this case, the approach requires sampling from distributions of the coefficients defining the exposure-response relationships for each age/sex group and area, following a recently proposed method [10].

## 3 Case-study illustration of the proposed framework

### 3.1 Background—UK Heatwave, 17–19 July 2022

During 17–19 July 2022, the United Kingdom experienced one of the most intense heat waves ever documented [15]. The UKMO released the first heat health warnings on 5 July, and a national emergency was declared on 15 July when temperatures were predicted to reach a peak of over 40 °C during the heatwave period, surpassing previous records and prompting the issue of the first-ever level 4 heat-health alert (HHA) and Red National Severe Weather Warning Service (NSWWS) Extreme Heat warning [16].

**Figure 2:**
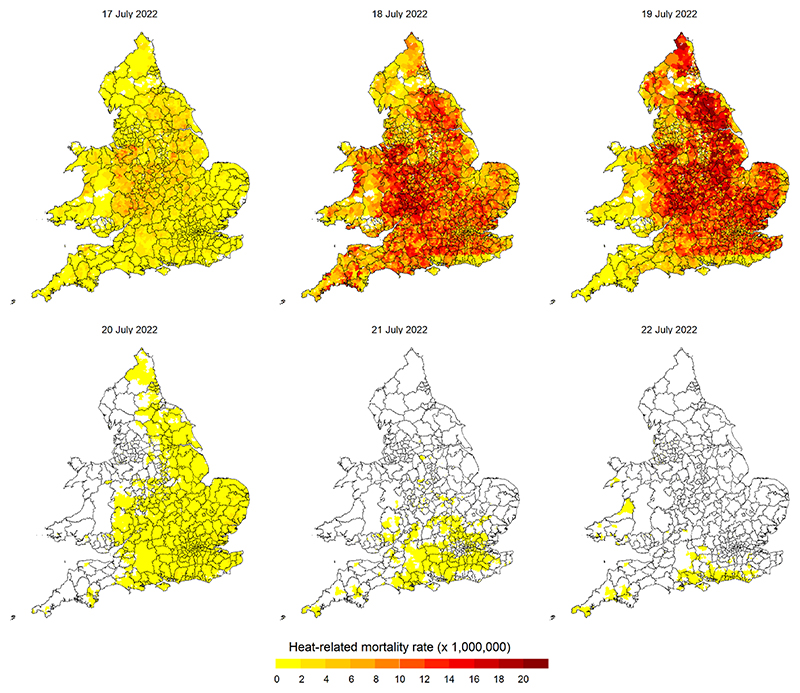
Maps of expected excess mortality rates (per 1000 000 people) by lower super output area (LSOA) for temperatures experienced during and after the heatwave of 17–19 July 2022 in England and Wales.

To demonstrate a practical application of the conceptual design and health impact forecast framework, we predicted the excess deaths attributable to heat that would have been expected across England and Wales during this 2022 heatwave and a few days following it. Put differently, we forecast the likely health impact of the July 2022 heatwave in the UK on a lead-time of six days (figure 2), spanning 17–22 July 2022, mimicking a forecast made in a real-time framework before the onset of the event. Our predictions are made at a geographic level of the Lower layer Super Output Areas (LSOAs), which are small census-based zones in England and Wales typically comprising an average population of about 1500, elaborated further in the next section.

### 3.2 Data

The estimation of age-specific excess deaths is based on local risk functions obtained in previous work [10]. Briefly, the estimation framework builds upon historical age-specific exposure-response relationships with a lag period of 0–21 days for each of the 37 473 LSOAs of England and Wales [10, 11]. These were then matched here with the 0.4° gridded forecasts of sub-daily mean temperature available from the European Centre for Medium-Range Weather Forecasts (ECMWF) Open Data [17], for the period 17–22 July 2022. Using information retrieved from National Online Manpower Information Service (NOMIS)—Official Labour Market Statistics, we then computed the expected number of excess deaths from a baseline calculated using age-stratified regional mortality rates and populations at each LSOA [12]. Additional information on data resources and related links are provided in the appendix.

### 3.3 Predicted excess deaths

Figure 2 shows the all-cause excess mortality rate across LSOAs for the temperatures forecast on the days 17–22 July 2022. Estimates aggregated by region, age group, and date are reported in table S1 in the appendix. The total number of expected excess deaths associated with the heatwave (17–19 July) was 1064 (95% eCI:735–1256), occurring mostly because of extreme temperatures on 18 and 19 July. This three-day excess is higher than the average heat-related excess mortality of 791 occurring each year in the period 2000–2019 [10]. The expected burden was particularly high among the elderly, with 589 (95%eCI:432–709) excess deaths in people aged 85 or older, indicating that about 55% of the expected total excess deaths could occur in an age group that makes 2.5% of the population (table S1).

The maps in figure 2 show that the impacts were highly heterogeneous across the geographic area, with higher excess mortality in the central regions. The highest excess of 26.9 deaths (95%eCI:19.5–31.2) for every 1000 000 people was expected in West Midlands, followed by East Midlands (21.6), East of England (20.2), and Yorkshire and The Humber (19.4). The lowest excess rates of 12.9 and 11.8 per 1000 000 people were expected respectively in the North–East and Wales (table S1 and figure S2). In addition, the impacts show a differential temporal variability across regions, with Cornwall (in the South-West Peninsula of England) and Wales more affected on 18 July, differently from the rest of the area that shows higher excesses during 19 July.

### 3.4 Estimates of excess deaths released by UKHSA-ONS

Though the July 2022 UK heatwave is used as an illustrative case study, we can now take advantage of the official estimated excess deaths for the same event to gauge the suitability of our framework as a real-time warning tool. The updated 2022 heat mortality monitoring report dated 10 July 2023, released jointly by the UKHSA and the UK Office for National Statistics (ONS) [7] almost a year after the heatwave event, is the most recent at the time of writing this study.

Our estimates of 1064 (95% eCI: 735–1256) total excess deaths, align well with the officially released number of 1256 (95% CI: 729–1784). Moreover, a quick comparison of our predictions stratified by age group and regions (table S1 in the Appendix) with the corresponding official estimates (table 2 in [7]) also reveals similar agreement between the two. A systematic comparison between our predictions and the official estimates is provided in table S2 for reference.

It should be noted that some dissimilarities with the official estimates can be due to the different study periods. The UKHSA-ONS estimates are aggregated over 10–25 July 2022, defined as Episode 2 -E2- in [7]. These official estimates therefore cover 16 days in contrast to three days over 17–19 July in our predictions as discussed in section 3.3.

Further, it is worth highlighting a few other crucial points here before elaborating on the same in section 4.2. First, our predictions of excess deaths are to be interpreted as ‘expected’ excess deaths and not ‘actual’ excess deaths, as the latter cannot be directly observed. Second, we deliberately chose to use forecast temperature data issued prior to the 2022 heatwave event, as well as existing risk functions and baseline mortality data, for predicting excess deaths. This allowed us to demonstrate a practical application scenario wherein the epidemiological modelling framework would be coupled with the publicly available meteorological forecast data prior to the event, and not take deceitful advantage of the (pseudo-) observations publicly available post hoc through climate reanalysis and station records. While benchmarking our predictions was not intended as the main motivation in this study, the close alignment of our estimates with the eventual official reported excess deaths can make one take note of the potential of the proposed framework.

## 4 Discussion

### 4.1 Key benefits of the proposed framework

Short-term temperature-related health impacts are often preventable if the associated health risks can be anticipated in advance and the susceptible population is forewarned to take appropriate preventive measures. The framework described here would facilitate real-time estimation of the expected temperature-related health effects based on geographically stratified risk functions and publicly available temperature forecasts, thus optimising the allocation of public health resources. It also has the potential to be developed as an automated environmental health-based warning system that can not only be used to direct local interventions, but also to assess their effectiveness when actual data on the health impacts become available.

Using high-resolution weather forecasts made publicly available by ECMWF, we applied state-of-the-art epidemiological methods described in previous studies [10, 11, 13] to illustrate a real-time application of predicting health impacts from an extreme temperature event, specifically the heatwave of 17–19 July 2022 in the UK. The underlying epidemiological model discussed in our setting is specific for assessing temperature-related mortality risks at local level. The framework, however, can not only be adapted to different geographical scales (e.g. municipalities, districts or counties), but can also be extended to other exposures (e.g. cold, humid-heat, air pollution) at different lead times. The conceptual framework itself is therefore flexible and scalable as per user requirements. Moreover, the entire framework can be deployed as an automated real-time prediction system by coupling high-resolution global weather forecasts routinely disseminated by weather centres such as the European (ECMWF), UK (UKMO) and the US (NOAA-National Centre for Atmospheric Research) meteorological services.

### 4.2 Methodological limitations and future extensions

The proposed framework is based on state-of-the-art epidemiological methods and temperature forecasting models. However, some methodological aspects and limitations need to be considered and possibly addressed in future research.

First, while our health impact estimates closely align with the figures reported in the UKHSA-ONS report, the framework has not undergone a thorough validation process, for instance by comparing excess deaths that occurred during various heat wave events in the past. In addition, the model assumes that exposure-response functions estimated in the historical period are representative of current risks, although there is evidence of temporal changes and attenuation in susceptibility to heat in recent decades [18]. In this case, local risk functions can be updated in the future with outputs of more advanced epidemiological methods that incorporate changes in susceptibility, for instance through adaptation.

A limitation is represented by the use of annual rates to derive baseline mortality, thus obtaining constant daily series with no seasonal trend. While limited and possibly involving a degree of additional uncertainty or bias, this method allows the application of the forecasting framework even in areas where observed daily mortality is not available, for instance in low and middle-income regions, adapting it to data and analyses performed at various geographical scales.

Another methodological issue is related to the potential differences in the data resources used for the estimation of the risk functions and the temperature forecasts. In our example, these were represented by the 1 × 1 km output from the Met Office’s HadUK-Grid database and the 0.4° × 0.4° forecast from the ECMWF [17]. A calibration step can be added to re-align the two sources and remove potential biases, using previously proposed methods [19].

Finally, it is worth noting that the uncertainty reported by the eCI only accounts for the uncertainty in the epidemiological model, and not the part related to the temperature forecast. One potential solution is to perform analyses using ensembles of forecasting models, similar to health impact assessments performed to quantify impacts under climate change scenarios [20].

### 4.3 Recommended practice for operational alerts

More generally, as highlighted in section 3.4, it is important to emphasise that the output of the epidemiological component is the ‘expected’ excess deaths. Given that the actual mortality burden can be different following the warnings and emergency policies implemented before and during an event (here heatwave), the predicted burden should not be construed as an estimate of observed mortality, but as a projected impact under specific assumptions. While it is important to validate the estimates post hoc against observations when implementing such a framework, its output can still provide relevant information to quantify potential benefits of interventions and allocate resources appropriately.

Equally important to emphasise is that data on the actual number of deaths are usually available only after several months following an event. Often such data are at a sub-national or national level, and they lack the finer-scale geographical disaggregation (as can be noted from the UKHSA-ONS report [7]), in contrast to our demonstrated application at small geographic levels such as the LSOAs. More importantly, these data also invariably refer to the total number of deaths, and not the excess associated with the specific event, though this is not the case with the UKHSA-ONS report referred here.

In summary, excess deaths can only be correctly estimated using models with varying degrees of sophistication and assumptions, such as the approach outlined here in our estimation. Other simpler approaches are available for the estimation of excess deaths, such as a comparison of deaths with those recorded in previous weeks or previous years. However, these are prone to biases from seasonal or long-term trends or concurrent extreme events, respectively, and cannot be stratified at small geographical scales. In contrast, our framework is based on state-of-the-art epidemiological designs and statistical methods that allow a fine and precise reconstruction of local risk functions and the generation of related excess mortality estimates, accompanied by uncertainty measures.

Several pitfalls and challenges in attributing observed deaths to a specific weather extreme exist and we refer the readers to a blog article by the ONS that nicely articulates some of them [21].

## 5 Conclusions

To conclude, the comprehensive framework outlined here has the potential to become a gold standard method for forecasting temperature-related excess mortality at a small-area level. When operationalised as a health alert system in real-time, the benefits of such an infrastructure are envisaged to improve planning and allocation of public health services, especially when health resources to cater to the vulnerable population are limited.

## Supplementary Material

Appendix

## Data Availability

The data and code that support the findings of this study are openly available at the following URL/DOI: https://github.com/gasparrini/UK-HWfcast.

## Data and code sharing

Original data resources and repositories are listed in the appendix. The ECMWF gridded temperature data from the 0.4° forecast was downloaded and processed using the open-source Python package ‘ecmwf-opendata’ (https://github.com/ecmwf/ecmwf-opendata). LSOA-specific risk functions representing the temperature-mortality exposure relationships were estimated in previous work [10], and retrieved using the original model parameters and associated data available in the GitHub repository. All the health impact analyses and output generation were done with the R software (version 4.2.3) [22]. The aggregation of the gridded temperature to the LSOA boundaries was done using package exactextractr (ver 0.10.0) [23]. Further details are provided in the Appendix. All maps were generated using R package ggplot2 (version 3.4.4) [24]. Figure 1 was made using the free online diagram software draw.io (https://app.diagrams.net/).
