# Supplementary material for: Real-time forecast of temperature-related excess mortality at small-area level: towards an operational framework
Source: Environ Res Health. Author manuscript; Available in PMC 2024 Aug 8. (PMC7616349; doi:10.1088/2752-5309/ad5f51)
Supplement: Appendix [file EMS197917-supplement-Appendix.pdf]

Supplementary appendix

Real-time forecast of temperature-related excess mortality at small-area level: towards an operational framework

Malcolm N. Mistry[1,2], Antonio Gasparrini[1]

[1]Environment & Health Modelling (EHM) Lab, Department of Public Health Environments and Society, London School of Hygiene & Tropical Medicine, London, UK
[2]Department of Economics, Ca' Foscari University of Venice, Venice, Italy

Corresponding authors: Antonio Gasparrini (antonio.gasparrini@lshtm.ac.uk) and Malcolm N. Mistry (malcolm.mistry@lshtm.ac.uk), Department of Public Health Environments and Society, London School of Hygiene and Tropical Medicine (LSHTM),15-17 Tavistock Place WC1H 9SH London, UK

Table of Contents **Page**

Data sources	2
Supplementary Figures and Tables	4
Figure S1	4
Figure S2	5
Table S1	6
Table S2	7
References	8

Data sources

The computation and mapping of the expected number of excess deaths was performed for each lower super output area (LSOA) in England and Wales. The LSOAs are census-based statistical units with approximately 1,500 residents. These areas correspond to the definitions used in the 2011 census, with a total of 34,753 LSOAs in the two countries. The computation was applied separately by age groups, specifically 0-64, 65-74, 75-84, and 85 and older. The data sources are described below.

(i) Temperature-mortality relationship: An exposure-response relationship is defined as a function that represents the risk across the exposure range using a specific reference value. In this context, exposure-response relationships are represented by non-linear dependencies that inform about the risk of mortality for all causes associated with daily mean temperature (°C). The risk usually increases at both high and low temperatures, with the lowest value corresponding to the minimum mortality temperature (MMT) used as a reference. Age-specific exposure-response relationships with a lag period of 0-21 days for all the LSOAs were estimated in a previous small-area study [1], using individual mortality records and temperature data for the period 2000-2019. Mortality data were provided by the Office of National Statistics (ONS, agreement MRP 2291/2013), while daily mean values of near-surface air temperatures on a 1x1 km grid across the United Kingdom were extracted from the HadUK-Grid database developed by the Met Office [2]. Specific details are provided in the original publication [1].

(ii) Forecast temperatures were retrieved from the 3-hourly '2 metre temperature' (variable '2t') covering the period 17/07/2022 (03h UTC) - 22/07/2022 (24h UTC) from the ECMWF high-resolution Open Data [3]. The data are provided on a 0.4°x 0.4° spatial grid (~45km) and based on medium-range high-resolution forecast models (HRES). These data are made publicly available by the ECMWF as part of their real-time meteorological products. The single-level temperature data in grib2 format (https://www.ecmwf.int/en/forecasts/datasets/set-i#I-i-a_fc, '2t', unit: °K) were accessed from the forecast issued on 17/07/2022 at 00h UTC using the open-source python package 'ecmwf-opendata' (https://github.com/ecmwf/ecmwf-opendata). We next aggregated the 3-hourly gridded temperature fields to daily averages in °C in Python, and then spatially aggregated them to the 37,473 Lower Super Output Areas (LSOAs) in England and Wales. The spatial aggregation to LSOA boundaries was performed in R (version 4.2.3) [4] using the package 'exactextractr' (version 0.10.0, https://github.com/cran/exactextractr) [5], and polygon shape files from the Open Geography portal of ONS [6] (elaborated below). The LSOA-aggregated level forecast daily temperature for 17-22 July 2022 were finally matched to the estimates of age-specific exposure-response functions between temperature and all-cause mortality obtained from the earlier published analysis [1].

(iii) Baseline daily mortality counts were estimated by applying age-stratified mortality rates to populations in each LSOA. Both sources of information were retrieved from the latest updated data

available from the National Online Manpower Information Service (NOMIS) – Official Labour Market Statistics (https://www.nomisweb.co.uk/; accessed last on 15-January-2024). Specifically, we used annual mortality rates for 2022 corresponding to the nine administrative regions of England plus Wales, applied to age-specific population sub-groups in each LSOA estimated in 2020. The use of regional rates, although cannot capture local differentials in mortality, avoids problems with highly imprecise estimates obtained at the LSOA level. It must be noted though that in a practical scenario, the data on small-area population and mortality rates provided by the local authorities may not be the most recent at the time of a weather event. Nevertheless, both population and mortality rates generally remain stable in the recent preceding years, and our sensitivity checks using population and mortality rates data for 2020 in lieu of 2022 do not affect the overall results.

(iv) **Geographical boundaries** of census administrative areas were defined by shapefiles retrieved from the Open Geography Portal (https://geoportal.statistics.gov.uk/) of the Office of National Statistics (ONS) [6]. The boundaries refer to both LSOAs and local authority districts (LADs) and are consistent with the definition in the 2011 census. This information was used to assign LSOA-specific forecasted temperature series and to create maps using GIS methodologies.

Supplementary Figures and Tables

Figure S1. Difference between forecast temperatures during and after the heatwave of 17-19 July 2022 and the maximum mean daily temperature recorded within the period 2000-2019, by lower super output area (LSOA) in England and Wales.

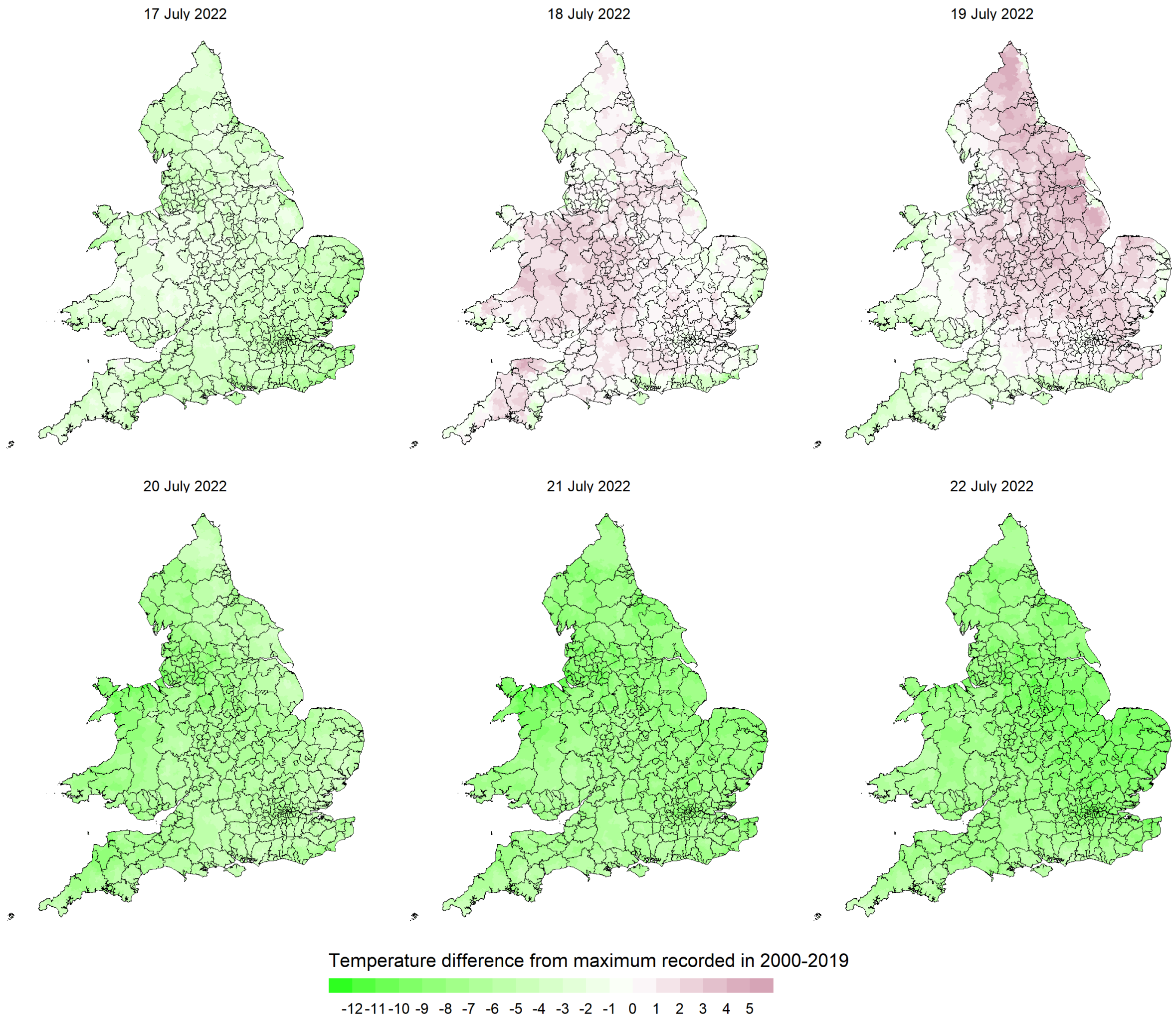

Figure S2. Regions in England and Wales used for aggregating population and excess-deaths in Tables S1 and S2.

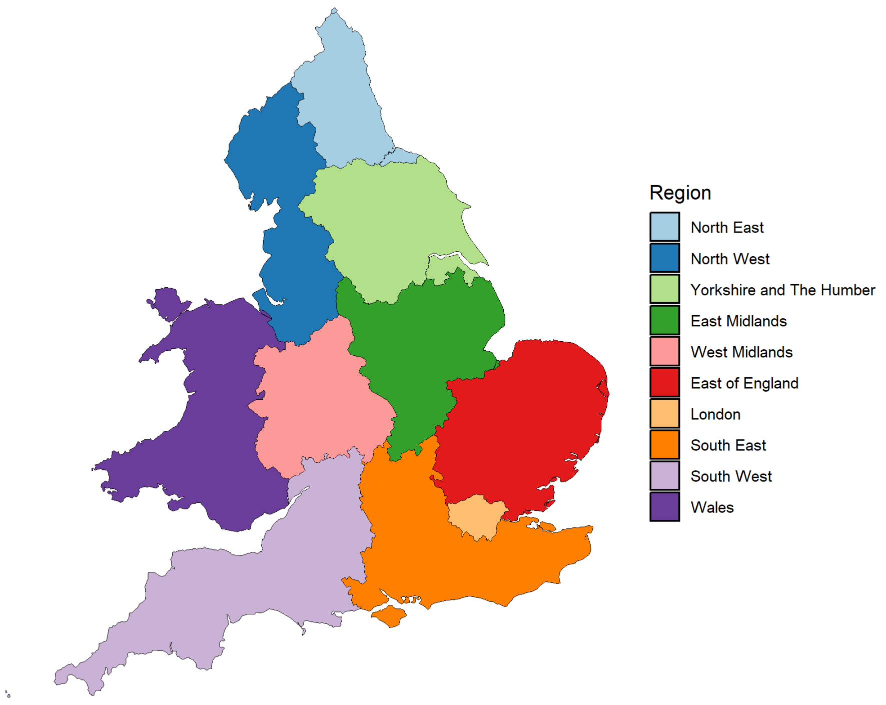

Table S1. Number of expected excess deaths and rate (per 1,000,000 people) (95% eCI) in England and Wales stratified by region, age group, and date, as predicted during the heatwave of 17-19 July 2022.

	**Population**	**Excess deaths**	**Excess death rate (per 1,000,000 people)**
**Region**			
East Midlands	4,865,583	105 (62 to 134)	21.6 (12.7 to 27.5)
East of England	6,269,161	127 (86 to 152)	20.2 (13.8 to 24.2)
London	9,002,488	153 (119 to 179)	17.0 (13.2 to 19.9)
North East	2,680,763	35 (-15 to 63)	12.9 (-5.6 to 23.7)
North West	7,367,456	105 (61 to 135)	14.2 (8.3 to 18.3)
South East	9,217,265	152 (102 to 188)	16.5 (11.1 to 20.4)
South West	5,659,143	82 (31 to 109)	14.4 (5.5 to 19.3)
Wales	3,169,586	37 (6 to 58)	11.8 (1.9 to 18.2)
West Midlands	5,961,929	160 (117 to 186)	26.9 (19.5 to 31.2)
Yorkshire and The Humber	5,526,350	107 (55 to 139)	19.4 (9.9 to 25.1)
**Age group**			
0-64	48,587,115	148 (18 to 234)	3.0 (0.4 to 4.8)
65-74	5,960,269	85 (-67 to 176)	14.2 (-11.3 to 29.6)
75-84	3,680,770	243 (92 to 361)	66.0 (25.0 to 97.9)
85+	1,491,570	589 (432 to 709)	394.6 (289.5 to 475.6)
**Date**			
17-Jul-22	59,719,724	117 (82 to 146)	2.0 (1.4 to 2.4)
18-Jul-22	59,719,724	453 (313 to 538)	7.6 (5.2 to 9.0)
19-Jul-22	59,719,724	494 (335 to 575)	8.3 (5.6 to 9.6)
**Total (all regions and age groups)**			
**17-Jul-22 to 19-Jul-22**	59,719,724	1,064 (735 to 1,256)	17.8 (12.3 to 21.0)

Table S2. Comparison of predicted expected excess deaths (95% eCI) and UKHSA-ONS official estimates (95% CI) by age groups and regions in England and Wales.

Note: The predicted excess deaths are for 17-19 July 2022. The UKSHA-ONS estimates are extracted from Tables 1 and 2 in ref [7] and instead cover 10-25 July 2022.

	**Population**	**Expected excess deaths (17-19 July 2022)**	**Official excess deaths (10-25 July 2022)**
**Region**			
East Midlands	4,865,583	105 (62 to 134)	96 (-48 to 239)
East of England	6,269,161	127 (86 to 152)	144 (-10 to 298)
London	9,002,488	153 (119 to 179)	184 (19 to 349)*
North East	2,680,763	35 (-15 to 63)	8 (-119 to 135)
North West	7,367,456	105 (61 to 135)	30 (-167 to 227)
South East	9,217,265	152 (102 to 188)	270 (33 to 507)*
South West	5,659,143	82 (31 to 109)	101 (-78 to 281)
Wales	3,169,586	37 (6 to 58)	NA
West Midlands	5,961,929	160 (117 to 186)	193 (17 to 369)*
Yorkshire and The Humber	5,526,350	107 (55 to 139)	236 (63 to 409)*
**Age group**			
0-64	48,587,115	148 (18 to 234)	68[#]
65-74	5,960,269	85 (-67 to 176)	104 (-108 to 316)
75-84	3,680,770	243 (92 to 361)	272 (-13 to 557)
85+	1,491,570	589 (432 to 709)	813 (481 to 1,144)*
Total	59,719,724	1,064 (735 to 1,256)	1,256 (729 to 1,784)

* Statistically significant.

[#] Aggregated using reported excessed deaths over age groups 0-24, 25-44 and 45-64. CIs are therefore not reported in brackets.

References

1. Gasparrini A, Masselot P, Scortichini M, et al (2022) Small-area assessment of temperature-related mortality risks in England and Wales: a case time series analysis. Lancet Planet Health 6:e557–e564. https://doi.org/10.1016/S2542-5196(22)00138-3
2. Met Office, Hollis D, McCarthy M, et al (2021) HadUK-Grid Gridded Climate Observations on a 1km grid over the UK, v1.0.3.0 (1862-2020). NERC EDS Centre for Environmental Data Analysis, doi:10.5285/786b3ce6be54468496a3e11ce2f2669c. Accessed 19 Aug 2023
3. European Centre for Medium-Range Weather Forecasts ECMWF Open Data. https://www.ecmwf.int/en/forecasts/datasets/open-data. Accessed 15 Jul 2022
4. R Core Team (2020) R: A Language and Environment for Statistical Computing
5. Daniel Baston (2023) exactextractr: Fast Extraction from Raster Datasets using Polygons R package version 0.10.0, <https://CRAN.R-project.org/package=exactextractr>.
6. Office for National Statistics (ONS) Open Geography Portal. https://geoportal.statistics.gov.uk. Accessed 4 Sep 2023
7. UK Health Security Agency (UKHSA), Office for National Statistics (ONS) (2022) Heat mortality monitoring report: 2022 (Updated July 10, 2023), https://www.gov.uk/government/publications/heat-mortality-monitoring-reports/heat-mortality-monitoring-report-2022. Accessed 22 December 2023
